# Overcoming Endocrine Resistance in Hormone-Receptor Positive Advanced Breast Cancer-The Emerging Role of CDK4/6 Inhibitors

**DOI:** 10.23937/2378-3419/2/4/1029

**Published:** 2015-10-14

**Authors:** Ciara C O’Sullivan

**Affiliations:** Medical Oncology Branch, Center for Cancer Research, National Cancer Institute, USA

**Keywords:** CDK4/6 inhibitors, Palbociclib, Ribociclib, Abemaciclib, Endocrine resistance, Breast cancer

## Abstract

Dysregulation of the cyclin D and cyclin-dependent kinase (CDK) pathway in cancer cells may inhibit senescence and promote cellular proliferation. By using various different mechanisms, malignant cells may increase cyclin D-dependent activity. The cyclin D-cyclin-dependent kinases 4 and 6 (CDK4/6)-retinoblastoma (Rb) pathway controls the cell cycle restriction point, and is commonly dysregulated in breast cancer; making it a rational target for anticancer therapy. To date, three oral highly selective cyclin-dependent kinase 4/6 inhibitors (CDK4/6i) are in various stages of clinical development: PD0332991 (palbociclib), LEE011 (ribociclib) and LY2835219 (abemaciclib). Results from phase I, II and III trials in hormone-receptor (HR)-positive breast cancer have been encouraging, demonstrating convincing efficacy and a tolerable side-effect profile (mainly uncomplicated neutropenia). This article will review the preclinical and clinical development of the CDK4/6i, as well as reviewing the existing preclinical evidence regarding combination of these agents with chemotherapy and other targeted therapies. Future and ongoing clinical trials, which may expand the potential application of these agents, will also be discussed. In summary, CDK4/6i are exciting compounds which may change the therapeutic landscape of HR-positive breast cancer.

## Introduction

Despite advances in treatment, metastatic breast cancer (MBC) is still incurable, and approximately 40,290 women will die of this disease in the United States in 2015 [1]. Approximately three-quarters of patients with MBC have hormone receptor positive disease (estrogen receptor positive [ER+] and/or progesterone receptor positive [PR+]) [2]. In the absence of a visceral crisis, endocrine therapy is the cornerstone of treatment for patients with human epidermal growth factor /HER2-negative disease [3], however progression is inevitable and cures are rare due to the development of endocrine resistance [4]. Elucidating the mechanisms of endocrine resistance is a research priority and hopefully will assist in development of more effective treatments for these patients. Recently, there has been a renewed interest in combining chemotherapy with endocrine therapy to overcome this problem. The combination of the mammalian target of rapamycin (mTOR) inhibitor everolimus and the steroidal aromatase inhibitor (AI) exemestane was more effective than exemestane alone in postmenopausal women with ER-positive, HER2-negative MBC who had progressed on one line of prior endocrine therapy [5]. This observation prompted Food and Drug (FDA) approval of exemestane and everolimus in this setting in 2012 [6]. However, as this regimen is associated with toxicities such as stomatitis and diarrhea [7], development of minimally toxic regimens without compromising efficacy is of great importance.

Finn et al. reported that combination therapy with the cyclin-dependent 4/6 inhibitor (CDK4/6i) palbociclib and the non-steroidal AI letrozole was well tolerated and associated with disease response in postmenopausal females with hormone refractory ER-positive and HER2-negative MBC [7]. A subsequent interim analysis noted that progression free survival (PFS) doubled in patients on palbociclib and letrozole compared with letrozole monotherapy (20.2 months vs. 10.2 months, respectively) [8]. In February 2015, the FDA approved the combination of palbociclib and letrozole as initial endocrine-based therapy for postmenopausal females with ER-positive, HER2-negative disease [9]. More recently, the combination of palbociclib and the estrogen antagonist, fulvestrant, increased median PFS in women with advanced HR-positive, HER2-negative breast cancer who had progressed on prior endocrine therapy (9.2 months in the palbociclib and fulvestrant arm vs. 3.8 months in the placebo and fulvestrant arm (HR 0.422, 95% CI 0.318 – 0.560, P < 0.000001) [10]. Therefore, determining efficacy of CDK4/6i in combination with other types of endocrine therapy and in different clinical scenarios is currently an important topic in HR-positive MBC.

### CDK 4/6 inhibition and the cell cycle

**Figure 1:**
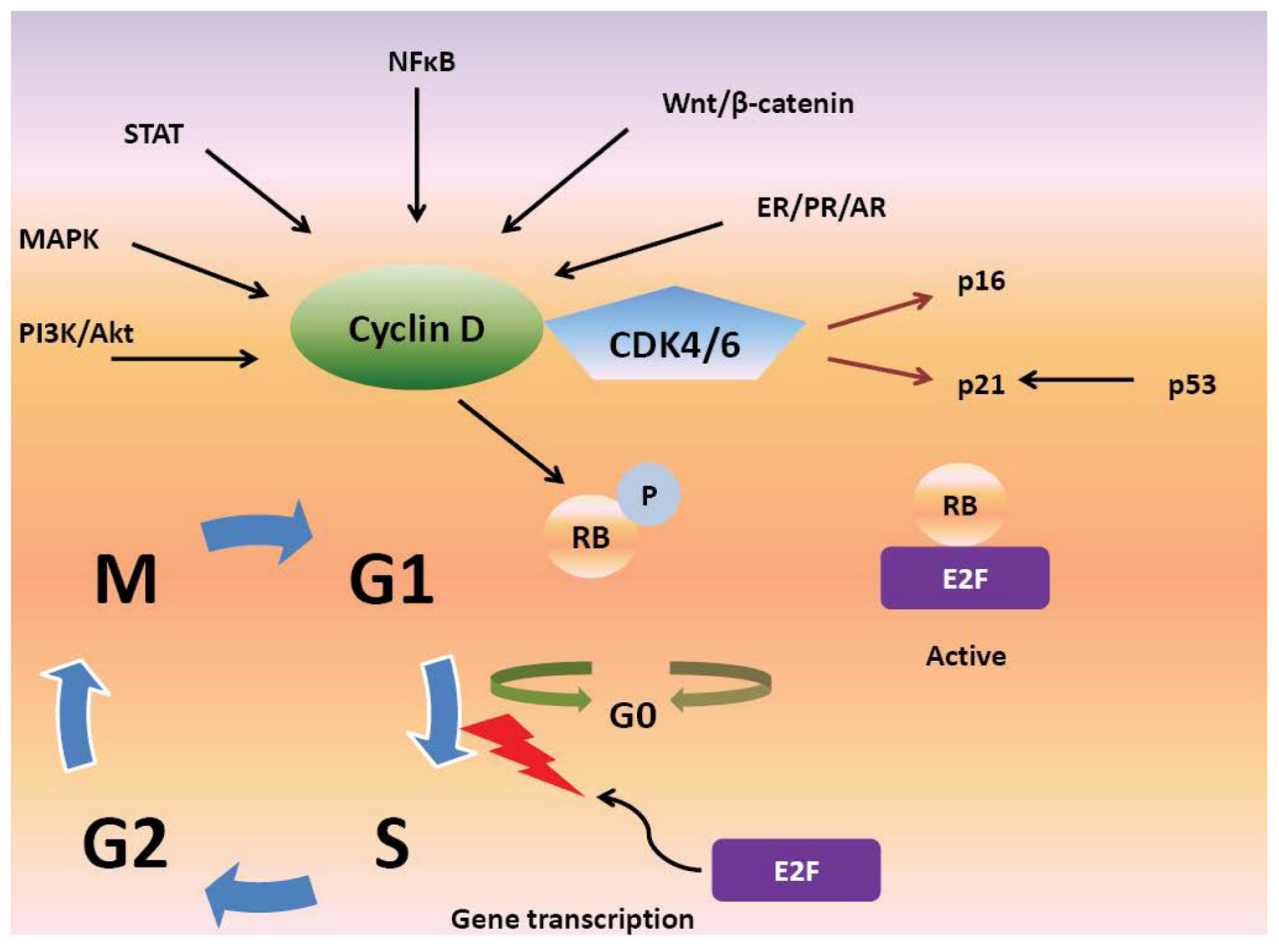
The role of Cyclin D, CDK4/6 and Rb in cell cycle progression. PI3K/Akt: phosphotidylinositol kinase/Akt, MAPK: mitogen-activated protein kinases, CDK4/6: cyclin-dependent kinase 4/6, NFκB: Nuclear Factor-Kappa B, ER: estrogen receptor, PR: progesterone receptor, AR: androgen receptor, RB: retinoblastoma, P: phosphate.

Imbalance between cellular senescence and proliferation is a classic feature of cancer [11,12]. When cells reach the “restriction point” during the G1 phase of the cell cycle, they decide whether or not to progress to S-phase, or rest in G0 phase [13]; a decision which is influenced by antigrowth signals. The cell cycle machinery mediates anti-proliferative signals from the extracellular environment by promoting transition from the G1 phase to the S phase [14]. These signals are then transmitted via the retinoblastoma tumor suppression protein (Rb), and related proteins p107 and p130. Cyclin-dependent kinases (CDKs) are a family of serine/threonine kinases, which interact with specific regulatory subunits called cyclins [15]. In order for cells to transition from G1 to S phase, Rb must be phosphorylated by CDK4 or CDK6 (often called CDK4/6 as they have similar mechanisms of action [16], and their activating cyclins (D1, D2 or D3) [17] (Figure 1). The CDK interacting protein/ kinase inhibitory protein (Cip/Kip) family also regulates formation of activated cyclin D-CDK4/6 heterodimers [18]. Phosphorylation and inactivation of Rb causes dissociation of E2F transcription factors and transcriptional regulation of genes which play an important role in facilitating G1-S phase transition, DNA replication, DNA repair and mitosis [19–24]. The cyclin-D/CDK4/6-Rb pathway, therefore, plays a key role in cell cycle regulation as it is downstream of multiple mitogenic cascades, making it an important target for drug development [25]. For example, treatment of endocrine-resistant breast cancer cell lines with the highly selective CDK 4/6 inhibitor palbociclib resulted in dephosphorylation of Rb and cell-cycle arrest, which was not seen when the cell lines were treated with fulvestrant [26]. This is particularly relevant given that there are many mechanisms of endocrine resistance, although not all of these are directly related to the cell cycle [27–31].

### Cyclin D-CDK4/6-Rb pathway dysfunction in breast cancer

Inactivation of the tumor suppressor and CDK inhibitor p^16INKA^, which can occur by several different mechanisms, is seen in approximately half of invasive breast cancers [32]. p16 inactivation is associated with uncontrolled cellular proliferation, de-differentiation and cellular proliferation [32]. Although p^16INKA^ is a tumor suppressor, overproduction of this protein has also been linked to disease progression [33]. Many tumors augment cyclin D-dependent activity, and avoid cellular senescence via multiple mechanisms, i.e. inactivation of p16, amplification of CDK4, mutation of CDK4 with loss of INK4 binding, overexpression of Cyclin D1, or via translocation or amplification of CCND1, its encoding gene [15]. Approximately 20–30% of breast cancers have loss of Rb expression, therefore the majority of breast cancers are Rb proficient [34]. Rb loss promotes tumor progression via loss of proliferation control and conversion to invasive disease and is more commonly seen in triple negative breast cancer (TNBC), where it portends a more favorable prognosis [35–37]. However, when Rb dysregulation is seen in ER-positive breast cancer, it is a poor prognostic indicator as this phenotype is associated with increased metastatic potential [38]. Although most breast cancers maintain functional Rb, a variety of other factors can corrupt the CDK4/6-cyclin D pathway, promoting cellular proliferation and tumor growth [39]. In Rb-proficient tumors, cyclin D1 levels limit cell division by influencing Rb phosphorylation and activation [40]. Cyclin D1 is a direct transcriptional target of ER, and injection of anti-cyclin D1 antibodies inhibits G1-S phase transition, which is estrogen dependent [41]. Cyclin D1 overexpression is seen in ≥ 50% of breast cancers, but this is of uncertain prognostic relevance [42,43]. Cyclin D1 amplification (CCND1) gene is seen in approximately 15–20% of breast cancers, and persists after the development of metastases [44–46].

High levels of cyclin E, resulting in Rb pathway dysfunction, cytoplasmic localization of p21 and p27Kip1 loss are common occurrences and are also associated with estrogen resistance and inferior breast cancer outcomes [29,47–49]. There is preclinical evidence which links Rb inactivation to tamoxifen and fulvestrant resistance [50], Further, there is preclinical and clinical evidence which shows that cyclin D1 overexpression promotes the formation of cyclin D1-CDK4/6 complexes, which in turn activate cyclin E1-CDK2 complexes and tamoxifen resistance [51–54]. Additionally, amplification of MYC inhibits p^21CIP21^ expression, which leads to formation of cyclin E1-CDK2 complexes and resistance to tamoxifen [55]. Less is known regarding mechanisms of resistance to AI therapy, however they probably differ to the mechanisms underlying tamoxifen and fulvestrant resistance in that cellular stress response activation and apoptosis are implicated [56].

### CDK4/6 inhibitors-preclinical and clinical development

Early studies with first-generation pan-CDK inhibitors such as flavoperidol were associated with minimal benefit and notable toxicities [57]. More recently, increasingly potent, selective small molecule CDK4/6 inhibitors have been developed [58]. Three of these agents are currently being evaluated in the clinical setting: PD0332991 (palbociclib), LEE011 (ribociclib) and LY2835219 (abemaciclib). Palbociclib is the furthest along in clinical development (Table 1).

### Palbociclib (PD0332991)

The orally active pyridopyrimidine palbociclib is a first-in-class potent, highly selective reversible inhibitor of CDK4 and CDK6. It has a molecular weight of 573.67 kDA and has a half-life of approximately 26 hours [59].

#### Mechanism of action

Over activation of the cyclin D1-CDK4/6 pathway is commonly seen in ER-positive breast cancer, and has a key role in promoting oncogenesis in this setting [60]. As the cyclin D1-CDK4/6-Rb complex acts as an estradiol effector, the actions of estradiol and G1-S phase transition are closely linked [47]. Palbociclib exerts its mechanism of action by impeding G1-S phase progression, and it has a cytostatic and cytoreductive effect on neoplastic cells [61], and it has been noted that injection of antibodies to cyclin D1 inhibits G1/S phase mediated transition. Reduced expression of cyclin D1 is also seen in the setting of ER-positive cells deprived of estrogen [62], whereas persistent cyclin D1 expression and phosphorylation of Rb are seen in endocrine resistant tumors [26]. CDK4/6 associated kinase activity confers cyclin D1 with oncogenic potential, and in turn, the main role of the latter is to promote CDK4/6 activity [41]. CDK4/6 inhibitors are most active in tumors with cyclin D1 overamplification and palbociclib is most effective in ER-positive breast cancer cell lines (both estrogen sensitive and estrogen resistant) [63]. Further, preclinical data showed palbociclib, either alone or combined with endocrine therapy, is very effective in overcoming endocrine resistance [64]. Therefore, there was considerable evidence to support further development of CDK4/6i in this setting.

#### Combination therapy

Most of the breast cancer cell lines which responded to palbociclib treatment were of the luminal ER-positive subtype, and the combination of tamoxifen and palbociclib was synergistic in ER-positive human breast cancer cell lines [65]. In hormone resistant breast cancer cell lines, palbociclib combined with either fulvestrant or letrozole was more effective than fulvestrant or letrozole monotherapy [10,65]. In HER2-overexpressing cell lines, palbociclib combined with trastuzumab was synergistic [64]. Additionally, sequential combination of palbociclib and a PI3-Kinase inhibitor resulted in a strong apoptotic response. In order to further evaluate these interesting findings, several clinical trials are planned or already open [66].

Potential antagonism between the cytostatic effects of CDK4/6 inhibition and the cytostatic effects of chemotherapy has been observed when palbociclib was evaluated in TNBC cell lines [67]. Single agent palbociclib demonstrated activity in genetically engineered mouse models of a HER2-positive, Rb-proficient breast cancer; however, combinations of palbociclib with carboplatin resulted in lower cytotoxicity than single-agent chemotherapy [39]. Further, continuous co-administration of palbociclib with paclitaxel reduced the cytotoxic properties of this chemotherapy agent. Of note, subsequent experiments with paclitaxel and doxorubicin observed that acute synchronization with CDK4/6i improves the cytotoxicity of these chemotherapeutic agents, highlighting the importance of timing when CDK4/6i are used as antineoplastic agents [67,68].

### Clinical development of palbociclib

Palbociclib was first evaluated in humans in an open-label dose-finding study in patients with Rb-proficient solid tumors or non-Hodgkin’s lymphoma [69]. Two separate schedules were studied: Schedule 2/1 (2 weeks on daily treatment/1 week off treatment) and schedule 3/1 (3 weeks on daily treatment/ one week off treatment. Thirty-three patients were enrolled on the 2/1 schedule, and palbociclib was administered at doses ranging from 100 mg to 225 mg daily. Two dose limiting toxicities (DLTs) were observed at the 225 mg/day dose: 1 case of grade 3 neutropenia, 1 case of grade 4 neutropenia and 1 case of grade 4 thrombocytopenia. The 200 mg/day dose was selected as the maximum tolerated dose (MTD); the only DLT was myelosuppression (especially neutropenia). Based on preclinical data, myelosuppression was an anticipated side-effect given the activity of CDK4/6i in cells which divide rapidly [70]. Of 31 patients available for response assessment, one patient with testicular cancer had a partial response (PR), and an additional 9 (29%) patients with different tumor types had stable disease (SD), which lasted over 10 treatment cycles for 3 patients). Forty-one patients were enrolled on schedule 3/1; patients were treated with daily doses ranging from 25 mg to 150 mg daily. Five patients had DLTs (three cases of grade 3 neutropenia and 2 cases of grade 4 neutropenia), and the recommended dose for phase II evaluation was set at 125 mg daily. Non-hematological side effects were generally low grade in both schedules; the most common were fatigue, diarrhea, nausea and constipation. Importantly, no clinically significant electrocardiographic QT interval changes were observed on either of the schedules. Of 37 patients on schedule 3/1 in whom response was evaluated, 13 (39%) patients had SD for longer than 2 cycles of therapy, and 6 patients achieved SD beyond 10 cycles of therapy (including a patient with Rb-proficient breast cancer). Schedule 3/1 was chosen for further clinical development as it demonstrated a superior efficacy and safety profile [70].

Single-agent palbociclib at a dose of 125 mg daily (3/1 schedule) was evaluated in a phase II trial in patients with Rb-positive advanced breast cancer [71]. The observed toxicities were similar to those seen in phase I evaluation in that the only grade 3 or 4 adverse events (AEs) recorded were neutropenia and thrombocytopenia. Of 37 patients enrolled, 2 of 28 (7%) evaluable patients had a partial response (PR), and 17 patients had SD, which lasted ≥ 6 months in 3 cases. In patients with TNBC, no responses or prolonged disease stabilization was noted. While palbociclib was more effective in patients with HR-positive and HER2-positive breast cancer, overall response rates were modest. The lack of efficacy of palbociclib monotherapy in this heavily pretreated population was attributed to potential cross-resistance with chemotherapy. Therefore, further evaluation of palbociclib in combination with other agents, including endocrine therapy, was planned.

The PALOMA-1 study (NCT00721409) was a multicenter, randomized phase I/II study, whereby postmenopausal patients with HR-positive, HER2-negative breast cancer were treated with palbociclib (at a dose of 125 mg orally daily on the 3/1 schedule) combined with letrozole 2.5 mg daily continuously versus letrozole alone [65]. Eligible patients received either regimen as first line treatment for metastatic disease. An initial phase I trial assessed the safety and tolerability of the combination, and enrolled patients on the basis of HR and HER2-status alone; however, in part 2 of the trial, the presence of CCND1 amplification and/or p16 loss was also needed for eligibility. Sixty-six patients were enrolled in the first part of the study, and 99 patients were enrolled in part 2. As the exploratory analysis showed that CCND1 status and p16 expression did not add additional predictive value for the efficacy of palbociclib over ER status alone, parts 1 and 2 of the trial were merged for a cumulative efficacy analysis. The first interim analysis of 66 patients had shown that the median PFS for the palbociclib and letrozole arm was 18.2 months vs. 5.7 months for the letrozole alone arm (HR = 0.35; 95% CI: 0.17, 0.72; p = 0.006) [7]. The second interim analysis of 165 patients were impressive, and PFS increased over three fold in patients treated in the combination arm (26.1 vs. 7.5 months; respectively; HR = 0.37; 95% CI: 0.21 – 0.63; p < 0.001) [65]. Once again, combination therapy was very well tolerated and the most frequent side effects were uncomplicated neutropenia (61.4% in patients treated with both agents compared with 1.3% in patients treated with single-agent letrozole), leukopenia, anemia and fatigue. On the basis of these favorable results, the FDA designed palbociclib as a “breakthrough therapy” for MBC in early 2015 [9].

An updated analysis of 100 PFS events was presented at the 2014 American Association for Cancer Research (AACR) meeting [8,65], and combination therapy with palbociclib maintained a striking improvement in PFS compared with the letrozole alone arm (20.2 months versus 10.2 months, HR 0.488, p = 0.0004). A significant difference in overall survival (OS) has not yet been noted, however a preliminary analysis was suggestive of a trend towards increased OS (37.5 months vs. 33.3 months, HR 0.813, p = 0.2105) [8]. Once again, the side effect profile for combination therapy was tolerable, and common grade 3/4 toxicities seen in the palbociclib containing arm vs. the letrozole alone arm were neutropenia (54% vs. 1%), leucopenia (19% vs. 0%), fatigue (4% vs. 1%) and anemia (6% vs. 1%). These promising results prompted Pfizer to submit a new drug application (NDA) to the FDA for combination therapy with palbociclib and letrozole as first-line treatment for postmenopausal women with ER-positive, HER2-negative advanced or MBC [72]. In February 2015, palbociclib was granted accelerated approval for the aforementioned indication by the FDA [9], however continued approval may be contingent upon verification and description of clinical benefit in the confirmatory phase III PALOMA-2 trial (NCT01740427). In this trial, eligible patients with previously untreated, advanced HR-positive, HER2-negative breast cancer are randomized to the combination of palbociclib and letrozole vs. letrozole alone. This study has enrolled 666 patients, and the estimated completion date is October 2015.

Results from the double-blind phase III PALOMA-3 trial (NCT0192135) were presented at the annual American Society of Oncology (ASCO) meeting in June 2015 [10]. This study evaluated the efficacy of palbociclib and fulvestrant in pre and postmenopausal women with HR-positive, HER2-negative advanced breast cancer who had relapsed or who had progressed on prior endocrine therapy. One prior line of chemotherapy for metastatic disease was permitted. Patients were randomized 2:1 to palbociclib (125 mg orally daily, 3 weeks on and one week of) and fulvestrant (500 mg I.M. per standard of care dosing) or to placebo and fulvestrant. Pre and peri-menopausal women were also treated with the luteinizing hormone releasing hormone (LHRH) agonist, goserelin. The primary endpoint was PFS and secondary endpoints included OS, safety and tolerability and patient-reported outcomes. At the first interim analysis, the primary endpoint was reached, and median PFS was 9.2 months in the palbociclib and fulvestrant arm and 3.8 months in the placebo and fulvestrant arm, (HR 0.422, 95% CI 0.318 to 0.560, P < 0.000001). The most frequent side effects on the palbociclib containing arm vs. the placebo-containing arm were neutropenia (78.8% vs. 3.5%), leucopenia (45.5% vs. 4.1%) and fatigue (38.0% vs. 26.7%). There was no increase in the febrile neutropenia in the palbociclib-containing arm (incidence 0.6% in both arms). Therefore, on the basis of these results, palbociclib and fulvestrant is a new treatment option for pre and postmenopausal patients with HR-positive, HER2-negative advanced breast cancer who have progressed on prior endocrine therapy [10].

Presently, several trials evaluating efficacy of palbociclib and endocrine therapy are in progress in the adjuvant (NCT02040857) and neoadjuvant settings (NCT02400567, NCT01723774, NCT01709370) in patients with HR-positive, HER2-negative breast cancer. For patients who do not achieve a pathological complete response (pCR) after neoadjuvant chemotherapy, NCT01864746 is an option. Combination therapy with chemotherapeutic agents such as paclitaxel is also being studied (NCT01320592), and NCT02028507 is a randomized phase III study comparing the efficacy and safety of palbociclib in combination with exemestane vs. capecitabine in patients with advanced ER-positive HER2-negative MBC, who have progressed on prior treatment with a non-steroidal aromatase inhibitor. Given the fact that preclinical data has noted that HER2-positive breast cancer cell lines (particularly those with luminal features), are sensitive to CDK4/6 inhibition, combinations with HER2-targeted agents are also being evaluated [73,74]. Adotrastuzumab emtansine, or T-DM1, is an antibody-drug conjugate which was approved for the treatment of HER2-positive MBC in 2013 [75]. As its mechanism of action appears to complement that of the CDK4/6i, there is particular interest in clinical evaluation of this combination, and a phase 1b study is currently recruiting participants (NCT01976169).

### Ribociclib (LEE011)

Ribociclib is an orally bioavailable, highly specific CDK4/6i which causes cell cycle arrest and tumor growth inhibition in multiple Rb-proficient preclinical models [76]. Additionally, ribociclib has shown dose-dependent antitumor activity which complements the mechanism of action of CDK4/6i [77]. In mouse models, ribociclib demonstrated single-agent activity in ER-positive breast cancers and/or activating mutations in HER2 and/or phosphotidyl-inositol kinase (P13KCA). Activity was also noted in melanomas with activating BRAF or NRAS mutations. Pharmacokinetic studies suggest that elimination of ribociclib may be affected by concomitant administration of medications which inhibit CYP3A4, CYP1A2 and BSEP [77]. NCT01237236 is a first in human, phase I trial, which is evaluating ribociclib in patients with advanced solid tumors or lymphomas. The projected enrollment is 160 patients and the estimated completion date is December 2016. As of January 2014, 132 patients had been treated on study. The most frequently observed side-effects were neutropenia (40%), leukopenia (36%), nausea (35%) and fatigue (27%) The most common grade 3/4 A/Es were neutropenia (19%), lymphopenia (14%) and leukopenia (12%), although most AEs were grade 1 or 2. At doses ≥ 600 mg, asymptomatic QTcF prolongations was noted more frequently, and the dose for phase II evaluation was set at 600 mg daily (3 weeks on/1 week off schedule) [76].

As per the ClinicalTrials.gov website, ribociclib is presently being studied in combination with everolimus and exemestane in patients with advanced HR-positive, HER2-negative breast cancer (NCT01857193). Preliminary results from this trial suggest that the triplet regimen is tolerable, and the phase II component is in progress [78]. Additional evaluation is ongoing in other solid tumors, including BRAF-mutated melanoma. As preclinical findings suggest that CDK4/6i helps overcome intrinsic and acquired resistance to PI3K inhibition [79], the combination of ribociclib with endocrine therapy and PI3-kinase inhibitors are also being evaluated in patients with advanced HR-positive, HER2-negative disease (NCT01872260, NCT02088684). Munster et al. presented results from their phase IB study evaluating the combination of ribociclib and letrozole (n = 10) and ribociclib and the PI3K inhibitor BYL719 [80]. Neutropenia was observed in the ribociclib arm as anticipated, but both combinations demonstrated acceptable safety profiles. Participants will be recruited to the third arm (ribociclib, letrozole and BYL719), followed by randomization to the latter regimen or either of the two doublets [57]. The phase II MONALEESA-1 trial (NCT01919229) was recently completed, this was a randomized preoperative study in patients with early stage HR-positive, HER2-negative disease in order to detect molecular changes in their tumors. The MONALEESA-2 trial (NCT01958021) is a randomized double-blind, placebo-controlled phase III study, which will evaluate the safety and tolerability of ribocliclib and letrozole in postmenopausal women with treatment-naive HR-positive, HER2-negative advanced breast cancer.

### Abemaciclib (LY2835219)

Abemaciclib is also a highly selective inhibitor of CDK4/6 that is in earlier stages of clinical development [81]. Preclinical data show that abemaciclib is a potent CDK4/6i, which inhibits phosphorylation of Rb, causing G1 arrest and inhibition of cellular proliferation [82]. Oral inhibition as monotherapy inhibits tumor growth in human tumor xenografts. Additionally, the combination of abemaciclib and gemcitabine increased *in vivo* tumor activity, albeit without G1 cell cycle arrest. These findings implied that abemaciclib could be used as monotherapy or combined with cytotoxic chemotherapy in Rb-positive tumors [81]. Single agent abemaciclib was studied in a phase I trial in patients with several tumor types at doses ranging from 50–225 mg daily and 75–275 mg daily, followed by an expansion phase in select tumor types (glioblastoma, melanoma and cancers of the lung, colon, rectum and breast) [83–85]. Fifty-five patients were enrolled on the expansion phase, where the MTD for every 12 hour schedule was 200 mg. DLTs were grade 3 fatigue at 200 mg (1/6 evaluable patients) and 275 mg (2/3 evaluable patients) [83]. In the MBC cohort, 47 heavily pretreated patients were treated with abemaciclib monotherapy. Nine patients had a PR (19%) and 24 patients had SD. Of the 36 patients with HR-positive disease, all of the 9 PRs were seen in this subgroup (9/36), with an overall response rate (ORR) of 25%. Additionally, 20 of these 36 patients (56%) had SD, and 13 of these 20 patients had SD ≥ 24 weeks [84]. The most frequent side-effects of treatment were diarrhea, nausea, fatigue, vomiting and neutropenia; the latter of which was the only grade 3/4 AE (in > 5% of patients). Subsequently, the combination of abemaciclib 200 mg twice daily and fulvestrant was evaluated in a separate cohort of patients with HR-positive MBC [85]. The most commonly observed treatment-related AEs were grade 3 neutropenia (33%), grade 3 leukopenia (23%), grade 3 diarrhea(8%), grade 3 fatigue (8%) and nausea/vomiting (grade 1& 2 only). Eight patients had a confirmed PR and 3 patients had an unconfirmed PR.

As per ClinicalTrials.gov, there are several open studies further evaluating the role of abemaciclib in patients with HR-positive breast cancer. The phase 2 MONARCH-1 (NCT02102490) trial is assessing the role of abemaciclib monotherapy in patients with previously treated, HR-positive, HER2-negative, advanced breast cancer, while MONARCH-3 (NCT02246621) is a randomized, double-blind, placebo-controlled trial of non-steroidal aromatase inhibitors with or without abemaciclib in women with advanced HR-positive, HER2-negative disease who have not had prior systemic therapy in this setting. MONARCH-2 (NCT02107703) is a randomized, double-blind placebo-controlled phase III trial which will compare abemaciclib at a dose of 200mg twice daily continuously combined with fulvestrant as per standard of care dosing versus fulvestrant alone, with PFS as the primary endpoint. NCT02057133 is a phase I study which is monitoring the safety of abemaciclib in combination with different standard endocrine therapies in patients with advanced HR-positive, HER2-negative breast cancer. Further, a non-randomized, phase II study of abemaciclib in patients with brain metastases secondary to HR-positive breast cancer is also recruiting (NCT02308020), and a neoadjuvant study of abemaciclib in postmenopausal women with HR-positive, HER2-negative breast cancer is also planned (NCT02441946).

## Conclusion/Discussion

Given the high incidence of recurrence and progression on standard therapies, developing new treatments for breast cancer is a research priority. As deregulation of the Cyclin-D-CDK4/6-Rb pathway is commonly seen in HR-positive breast cancer, this is a plausible target for drug development. While studies with first generation pan-CDK inhibitors were limited by modest clinical activity and substantial toxicity, treatment with more potent and selective CDK inhibitors have proven to be far superior in terms of efficacy and tolerability. Notably, in the randomized phase II trial of letrozole with or without palbociclib, PFS was doubled in the palbociclib-containing arm [86]. If results from the phase III PALOMA-2 trial are confirmatory, this will reflect a landmark change in the treatment paradigm for advanced HR-positive MBC.

While the manageable side effect profile and oral route administration of CDK4/6i and endocrine therapy will be attractive from a patient standpoint, combination therapy is much more expensive than endocrine therapy alone [87]. Additionally, the potential inconvenience to patients of extra blood draws and possible need for granulocyte-colony stimulation factor (GCSF) injections must be taken into consideration. Other factors, such as patient comorbidities, anticipated compliance with therapy and the burden of metastatic disease should also influence clinical decision making in this setting. Therefore, pharmaco-economic considerations and individualization of patient care are important, especially as data from PALOMA-2 is still awaited.

Ongoing research will evaluate the potential utility of CDK4/6i outside the arena of advanced HR-positive breast cancer. Adjuvant and neoadjuvant trials are currently in progress, and numerous studies in other solid tumor types are planned. Combination with chemotherapy has been problematic to date due to potential drug-drug antagonism, however this may be due to timing of drug administration in relation to chemotherapy. Of note, preclinical data suggest that cyclic palbociclib may be effective when combined with metronomic chemotherapy [65]. It will also be important to assess the clinical efficacy of palbociclib in combination with HER2-targeted therapy, mTOR inhibitors and PI3K inhibitors, and numerous clinical trials are ongoing or planned. The combination of everolimus and exemestane is approved for the treatment of HR-positive, HER2-negative MBC in patients who recurred or progressed on therapy with a non-steroidal AI [5], and the histone deacetylase inhibitor (HDACi) etinostat in combination with exemestane has also demonstrated activity in this setting [88]. Therefore, appropriate sequencing of these treatment options in the HR-positive MBC will need to be determined in the future. Interestingly, it has been observed that palbociclib can reverse epithelial dysplasia associated with abnormal activation of the cyclin D-CDK4/6-Rb pathway, which implies that this agent may potentially be useful as a chemopreventative agent [89]. Meanwhile, basic and translational research will attempt to detect predictive biomarkers in order to identify subgroups of patients that may be sensitive or resistant to treatment with CDK4/6i. Overall, CDK4/6i are exciting agents, and hopefully their potential to change the therapeutic landscape of HR-positive breast cancer will be borne out by the results of further phase III trials.

## Figures and Tables

**Table 1:** Important phase II & III trials of palbociclib in breast cancer.

Trial Name	Phase	Eligible patients	Disease setting	Number enrolled	Treatment regimen	Status/Results	NCT number or reference

**PALOMA-1**	I/II	Postmenopausal, ER+ & HER2− advanced BC	First line	165	Palbociclib^ & letrozole	PFS 20.2 months	[8]
					placebo & letrozole	PFS 10.2 months	

**N/A**	IIt	Rb-positive advanced BC	Prior treatment	28	palbociclib 125mg daily;	7% PR	[72]
					3 weeks on, 1 week off	14% SD	

**PALOMA-2**	III	Postmenopausal, ER+ & HER2− advanced BC	First line	450	palbociclib & letrozole	Ongoing, recruitment complete	NCT01740427
					placebo & letrozole		

**PALOMA-3**	III	Pre and Postmenopausal, ER+ & HER2− advanced BC*	Prior ET, 2nd line & beyond	521	palbociclib & fulvestrant	PFS 9.2 months	[10]
					placebo & fulvestrant	PFS 3.8 months	

N/A: Not applicable, ER+-Estrogen Receptor Positive, HER2: Human Epidermal Growth Factor Receptor Negative, Rb: Retinoblastoma Protein, BC: Breast cancer, ET: Endocrine Therapy, PFS: Progression Free Survival, SD: Stable Disease, PR: Partial Response, NCT: National Clinical Trials

* Pre and perimenopausal women received goserelin

^ Dosing of palbociclib was 125 mg orally daily; three weeks on and one week off.

Letrozole and fulvestrant were administered per standard care guidelines
